# S43. Dendritic cell vaccination combined with CTLA4 blockade

**DOI:** 10.1186/2051-1426-2-S2-I9

**Published:** 2014-03-12

**Authors:** K Thielemans, B Neyns

**Affiliations:** 1 Vrije Universiteit Brussel, Faculty of Medicine and Pharmacy, Brussels, Belgium; 2 University Hospital Brussels, Oncology, Brussels, Belgium

## Background

Electroporation of DC with mRNA also allows the loading of these cells with tumor antigens and the functional modification of the cellular vaccine. To this goal, we provide three different molecular adjuvants to immature, monocyte derived DCs through electroporation with mRNA coding for CD40L, CD70 and caTLR4 or so-called TriMix mRNA.

At our institution, clinical trials in pretreated advanced melanoma patients are being performed. These patients are treated with **TriMixDC-MEL**, a mixture of TriMix-DC co-electroporated with mRNA encoding a fusion of DC.LAMP and 1 of 4 melanoma associated antigens (gp100, tyrosinase, MAGE-C2 or MAGE-A3).

## Results

In a pilot clinical trial, 24.10^6^ TriMixDC-MEL cells were administrated solely by the intradermal (ID) route. Subsequently, a phase IB was conducted to investigate the safety of administrating TriMixDC-MEL by the intravenous (IV) and ID-route. ID administration of TriMixDC-MEL was found to be feasible, safe, effectively stimulating CD8^+^ T-cell responses, but did not result in objective tumor responses. In contrast, the combined ID/IV administration is associated with distinct but manageable side-effects and has seemingly superior clinical activity as compared to DC administered solely ID in patients with pretreated advanced melanoma. We also investigated the safety and activity of TriMixDC-MEL combined with ipilimumab. The best objective tumor response observed in this trial (37 evaluable pts) were 6 CR, 6 PR, 7 SD and 16 PD (disease control rate: 51%). All 6 CR and 3 PR are currently ongoing (respectively after 23, 22, 20, 20, 19, 17, 15, 14, 14 months). This phase II study of TriMixDC-MEL ID/IV in combination with ipi demonstrates anti-melanoma activity in over 50% of the patients with therapy resistant advanced melanoma.

## Conclusion

Further clinical development of TriMixDC-MEL in combination with immune checkpoint modulators is warranted.

Furthermore, TriMixDC-MEL is currently under evaluation in a randomized phase II trial in the adjuvant setting following resection of macrometastases.

